# Transcriptomic analysis reveals ethylene as stimulator and auxin as regulator of adventitious root formation in petunia cuttings

**DOI:** 10.3389/fpls.2014.00494

**Published:** 2014-09-26

**Authors:** Uwe Druege, Philipp Franken, Sandra Lischewski, Amir H. Ahkami, Siegfried Zerche, Bettina Hause, Mohammad R. Hajirezaei

**Affiliations:** ^1^Department of Plant Propagation, Leibniz Institute of Vegetable and Ornamental Crops (IGZ)Großbeeren/Erfurt, Germany; ^2^Department of Cell and Metabolic Biology, Leibniz Institute of Plant BiochemistryHalle, Germany; ^3^Institute of Biological Chemistry, Washington State UniversityPullman, WA, USA; ^4^Department of Molecular Plant Nutrition, Leibniz Institute of Plant Genetics and Crop Plant ResearchGatersleben, Germany

**Keywords:** adventitious rooting, wound, stress, transcriptome, plant hormones, Aux/IAA, ARF, ERF

## Abstract

Adventitious root (AR) formation in the stem base (SB) of cuttings is the basis for propagation of many plant species and petunia is used as model to study this developmental process. Following AR formation from 2 to 192 hours post-excision (hpe) of cuttings, transcriptome analysis by microarray revealed a change of the character of the rooting zone from SB to root identity. The greatest shift in the number of differentially expressed genes was observed between 24 and 72 hpe, when the categories storage, mineral nutrient acquisition, anti-oxidative and secondary metabolism, and biotic stimuli showed a notable high number of induced genes. Analyses of phytohormone-related genes disclosed multifaceted changes of the auxin transport system, auxin conjugation and the auxin signal perception machinery indicating a reduction in auxin sensitivity and phase-specific responses of particular auxin-regulated genes. Genes involved in ethylene biosynthesis and action showed a more uniform pattern as a high number of respective genes were generally induced during the whole process of AR formation. The important role of ethylene for stimulating AR formation was demonstrated by the application of inhibitors of ethylene biosynthesis and perception as well as of the precursor aminocyclopropane-1-carboxylic acid, all changing the number and length of AR. A model is proposed showing the putative role of polar auxin transport and resulting auxin accumulation in initiation of subsequent changes in auxin homeostasis and signal perception with a particular role of *Aux/IAA* expression. These changes might in turn guide the entrance into the different phases of AR formation. Ethylene biosynthesis, which is stimulated by wounding and does probably also respond to other stresses and auxin, acts as important stimulator of AR formation probably via the expression of ethylene responsive transcription factor genes, whereas the timing of different phases seems to be controlled by auxin.

## Introduction

Adventitious root (AR) formation is a developmental process which on the one hand reflects the great plasticity of plants to adjust to stressful environmental conditions and to regenerate plant structures on the same individual independent of sexual reproduction. On the other hand, this process is utilized in clonal plant propagation. In case of ornamentals this is carried out at industrial level and involves a complex global production chain providing several billions of rooted plants to the European market each year. Improvement of the understanding of the regulation of this developmental process should provide new tools and unravel starting points for improvement of propagation efficiency.

ARs are formed in stems, leaves and non-pericycle tissues of older roots (Li et al., 2009) and thus can be considered as being formed from cells of non-root pericycle identity. Tissues of origin are most frequently the cambium or adjacent vascular tissues, which undergo first mitotic divisions before either developing directly to root primordia or first showing a transient phase of callus formation (Li et al., 2009; da Costa et al., 2013). AR formation can be induced on intact plants according to the developmental program and in response to environmental factors such as stress factors. However, AR formation is particularly stimulated in excised plant parts (cuttings), where it is the combined result of responses to two stimulating principles: (i) wounding at the cutting site and (ii) isolation from the functional integrity of the whole plant, i.e., isolation from the support of signals and resources provided by the root system.

AR formation is a multistage process, of which the most widely recognized phases are induction, initiation and expression (Kevers et al., 1997; Li et al., 2009). The induction phase is devoid of any visible cell divisions and involves reprogramming of target cells to the following establishment of meristemoids, representing clusters of new meristematic cells (Kevers et al., 1997; De Klerk et al., 1999; da Costa et al., 2013). Based on studies with apple considering also the response to auxin pulse applications, De Klerk et al. (1999) established the concept of an early dedifferentiation phase occurring before the induction phase. However, since such a phase has not been proven as universal, we follow the concept of da Costa et al. (2013) and consider such possible events as early steps of the induction process. The following two phases (i) initiation and (ii) expression are characterized by (i) formation of root meristems and primordia and (ii) the establishment of vascular connections of the new structures to the original stem vascular system and the emergence of the roots from the stem (Kevers et al., 1997; Li et al., 2009). For simplification purposes these two phases have been joined under the single domination of formation phase (Fett-Neto et al., 1992; da Costa et al., 2013), which we also apply in this paper.

Among the diverse array of environmental and endogenous factors controlling AR formation plant hormones play an important role with an outstanding function of auxin (Kevers et al., 1997; De Klerk et al., 1999; Li et al., 2009; Pop et al., 2011). However, even though a substantial amount of work has been focused on these relationships, the knowledge is still fragmentary. Furthermore, the limited knowledge concerning the molecular control of AR formation is to a substantial extent based on work with *Arabidopsis* (Sorin et al., 2006; Ludwig-Müller, 2009; Gutierrez et al., 2012). Here, mostly hypocotyls of intact seedlings were used as source tissues usually leading to a formation of roots from pericycle cells. These contrast to root founding tissues in cuttings obtained from fully developed shoots (Correa et al., 2012; da Costa et al., 2013). In a recent update of main hormonal controls in AR formation, da Costa et al. (2013) pointed out that AR formation in cuttings is intrinsically tied to a stress response, which goes hand in hand with the developmental program. Integrating the fragments of knowledge obtained from different plant systems using different AR-inducing physiological principles and considering studies on primary or lateral root development, the authors developed a concept of possible phytohormonal interactions in AR formation. While auxin is considered as inductor of AR formation and as inhibitor of initiation of ARs, ethylene (ET), known to be in cross-talk with auxin, is assumed to act as stimulator of root expression. Cytokinins may stimulate very early processes of AR induction, but are inhibitory during the later phase of induction, while they are considered to be removed from the rooting zone by the transpiration stream shortly after excision. Strigolactones have inhibitory roles in AR formation (Rasmussen et al., 2012) and may directly inhibit initiation of AR or repress auxin action by reducing its transport and accumulation. Jasmonic acid (JA) is supposed to have dual functions as inducer of sink establishment in the rooting zone on the one side, and as negative regulator of root initiation on the other side (da Costa et al., 2013). Regarding diverse relations found between gibberellin (GA) application, GA-response and rooting (Busov et al., 2006; Steffens et al., 2006), GA may have a phase-dependent effect, being inhibitory to root induction but stimulatory to formation (da Costa et al., 2013). Due to reported negative effects on cell cycle progression (Wolters and Jürgens, 2009), on lateral root development in *Arachis hypogaea* (Guo et al., 2012) and on AR formation in rice (Steffens et al., 2006), ABA is thought to inhibit AR root induction (da Costa et al., 2013). On the other hand, ABA may protect plant tissues against abiotic stresses (Mehrotra et al., 2014).

The control and involvement of auxin homeostasis and of the intricate signaling network during AR formation still remain poorly understood (Ludwig-Müller, 2009; Pop et al., 2011). Therefore, a current model on these relationships is based on studies of primary and lateral root development and also other developmental processes (da Costa et al., 2013). As part of nuclear regulatory complexes, family members of the transport inhibitor response/auxin-signaling F-box (TIR/AFB)-complex proteins are considered to control the ubiquitination of Aux/IAA proteins via ubiquitin-protein ligases in dependence on auxin. Aux/IAA proteins bind to and thereby act as transcriptional repressors of ARFs (auxin response factors) (Tan et al., 2007; Chapman and Estelle, 2009). IAA acts via binding to TIR1/AFB and to Aux/IAA functioning as a glue, which allows ubiquitination and proteosomal degradation of the repressor Aux/IAA. This releases the ARF from repression, which then may act as activators or repressors on the transcription of auxin-responsive genes (Tiwari et al., 2003). In *Arabidopsis*, ARF6 and ARF8 have been identified as positive and ARF17 as negative regulators of AR formation (Gutierrez et al., 2012).

Microarray studies provide an ideal approach, to track complex regulatory pathways during plant development for detecting candidates of major control points and of linkages between different pathways. Considering the general features of *Petunia hybrida* as model plant (Gerats and Vandenbussche, 2005) and its high economic importance as vegetatively propagated plant, we developed *P. hybrida* as new model system to study molecular and physiological regulation of AR formation in shoot tip cuttings (Ahkami et al., 2009, 2013). Within this scope, a petunia microarray carrying approximately 25,000 unique, non-redundant annotated sequences has been established (Breuillin et al., 2010; Ahkami et al., 2014).

The objective of present study was to identify genes and related pathways putatively controlling the excision-induced reprogramming of particular cells to be newly determined as root meristems and developing further into root primordia and the complete body of the root. After microarray-based monitoring of gene expression during the process of AR formation, we focus on genes putatively involved in the change from stem to root identity. In particular, this analysis aimed to provide an overview on the involvement of the different instruments of the orchestra of plant hormone action during excision-induced AR formation in cuttings of *P. hybrida*. With regard to the important role of endogenous auxin for AR formation in this plant (Ahkami et al., 2013) and the strong response of ET-related genes, these two pathways will be discussed in detail and included in a model.

## Materials and methods

### Array screening and expression data analyses

Production of plant material, RNA extraction and array screening were described in Ahkami et al. (2009, 2014). Shortly, the stem base (SB) of leafy cuttings (*P. hybrida* cv. Mitchell) from 0 to 192 hours post-excision (hpe), fresh and wounded leaves (2 h after wounding) and a fully developed root system were used for RNA extraction. Three to four independent biological replicates were included per each time point and type of tissue. Probe synthesis, hybridization of microarrays and normalization of data was carried out by Nimblegene (Roche Nimblegene, Waldkraiburg, Germany). Depending on the length of the original sequence, per each gene three or two independent probes with an average length of 36 base pairs were spotted on the array. Normalized data were further used to calculate mean expression values. Significance of differences was calculated by Rank Product (RP) analysis running 1000 permutations (Breitling et al., 2004). Ratios of expression data obtained from different samples are presented as *M*-values (log_2_ of ratio). If *M-values* were > 1 or <−1 and RP values were <0.01, differences between samples were taken as significant. Each sequence identifier was annotated with one particular putative function based on manually curated similarity searches and classified into functional categories. The relative number of up- and down-regulated genes in each functional category was calculated by the following formula:

$$\frac{N^{{}^{\circ}}xp/N^{{}^{\circ}}xt}{N^{{}^{\circ}}ap/N^{{}^{\circ}}at}$$

*N*° *xp*, number of up- or down-regulated genes of a particular category; *N*° *xt*, number of all genes in the category; *N*° *ap*, number of all up- or down-regulated genes; *N*° *at*, number of all genes.

### Pharmacological experiments

Seedlings were germinated and grown under sterile conditions on agar containing half-strength MS medium (Klopotek et al., 2010). Roots of 2-weeks-old seedlings were removed and de-rooted plantlets were transferred on new agar medium supplemented with aminoethoxyvinylglycine (AVG), silver thiosulfate (STS), aminocyclopropane carboxylate (ACC), or ethephon at the concentrations indicated. Seedlings were cultivated vertically under long-day conditions at 22°C for 14 d. Root number and the average root length of treated plantlets were determined according to Klopotek et al. (2010) in comparison to plantlets grown on medium without the respective substance.

### Determination of ACC

About 0.5 g FW of homogenized plant material pooled from at least three cuttings was extracted with 10 ml methanol supplied with [^2^H_4_]-ACC as internal standard. The filtered homogenate was purified using DEAE-Sephadex A25 (Amersham Pharmacia Biotech AB, Uppsala, Sweden) and eluted by methanol. The eluate was evaporated, dissolved in 5 ml water and placed on a *LiChrolutRP-18*-column (Merck). The column was eluted with 2 ml of water. The evaporated eluate was dissolved in 200 μl CHCl_3_/*N,N*-diisopropylethylamine (1:1) followed by derivatization with 10 μl pentafluorobenzylbromide at 20°C overnight. After evaporation, samples were dissolved in 5 ml n-hexane and passed through a Chromabond-SiOH column (Machery-Nagel). The pentafluorobenzyl esters were eluted with 7 ml n-hexane/diethylether (2:1). Elute was evaporated, dissolved in 100 μl CH_3_CN and analyzed by gas chromatography mass spectrometry (GC-MS) as described by Miersch et al. (2008). All determinations were done at least from three independent biological replicates.

### Quantitative RT-PCR analysis

RNA was isolated from three biological replicative samples per time point. Determination of transcript accumulations of *PhACO1* was carried out by qRT-PCR as described previously (Ahkami et al., 2009) with three technical replications. Six different genes were tested as reference using geNorm v3.4, the Excel add-in of NormFinder v0.953, BestKeeper v1 and qBasePlus (Mallona et al., 2010). The gene for the cytoplasmic ribosomal protein S13 of *P. hybrida* (*PhCyRiPro*; CV2977) was finally selected as reference. Real-time PCR primers were designed using Primer Express software (Applied Biosystems, Warrington, UK). Primers used were *PhACO1*for:5′-TAC GTG CCC ACA CAG ATG C-3′, *PhACO1*rev:5′-GGG AGG AAC ATC GAT CCA TTG-3′, *PhCyRiPro*for: 5′-AAG CTC CCA CCT GTC TGG AAA-3′, *PhCyRiPro*rev: 5′-AAC AGA TTG CCG GAA GCC A-3′. Relative gene expression levels were calculated as 2^−ΔCT^ (ΔCT = CT_PhACO1_ − CT_PhCyRiPro_) using the MxPro QPCR-Software (Agilent Technologies, Waldbronn, Germany).

## Results

### Expression patterns during AR formation

During AR formation, particular cells in the SB undergo a developmental program from shoot to root identity. Transcript patterns were therefore recorded in the SB prior to excision, at different time points (2–192 hpe) and in a fully developed root system (see Table S3a in Ahkami et al., 2014). In addition, patterns were analyzed in fresh and wounded leaves to identify wound-associated genes and to distinguish those in the SB at the early stages (2 and 6 hpe) from wound-independent AR formation-regulated genes (see Table S3a in Ahkami et al., 2014). Based on these patterns, comparisons were conducted between samples at different developmental stages post-excision and the SB prior excision, between wounded and fresh leaves and between the three organs stem base, leaf and root (Table S1a). These comparisons showed a trend toward higher number of differentially regulated genes at later developmental stages post-excision (Table 1, columns 2 and 3; Figure S1A). This trend was even more pronounced, if wound-induced or wound-repressed genes were subtracted. To clarify this in more detail, the numbers of SB genes (Table S1b) and of root genes (Table S1c) were determined. These analyses showed that during AR formation an increasing number of stem base-expressed genes was repressed and of root-expressed genes was induced (Table 1, columns 4 and 5; Figure S1B).

**Table 1:** **AR formation-regulated genes**.

**Date**	**N° of AR formation-regulated genes**		**Stem base genes (in total 339) repressed during AR formation (values < 500)**	**Root genes (in total 476) expressed during AR formation (values > 500)**
	**All**	**(Minus wound-regulated)**		
2 hpe	5252	2709	63	116
6 hpe	6267	2490	191	132
24 hpe	5053	3417	118	135
72 hpe	6306	4673	278	275
96 hpe	5852	4338	197	224
144 hpe	5987	4542	243	279
192 hpe	6416	4832	304	314

*Columns two and three show the number of genes which are induced or repressed compared to the stem base before excision. For column four, stem base genes were defined as those showing expression values > 1000 in the stem base before excision and expression values < 100 in fully developed roots (Table S1b). For column 5, root genes are those with expression values > 1000 in fully developed roots and expression values < 100 in the stem base (Table S1c). A graphical presentation is shown in Supplemental Figure S1*.

**Figure 1:**
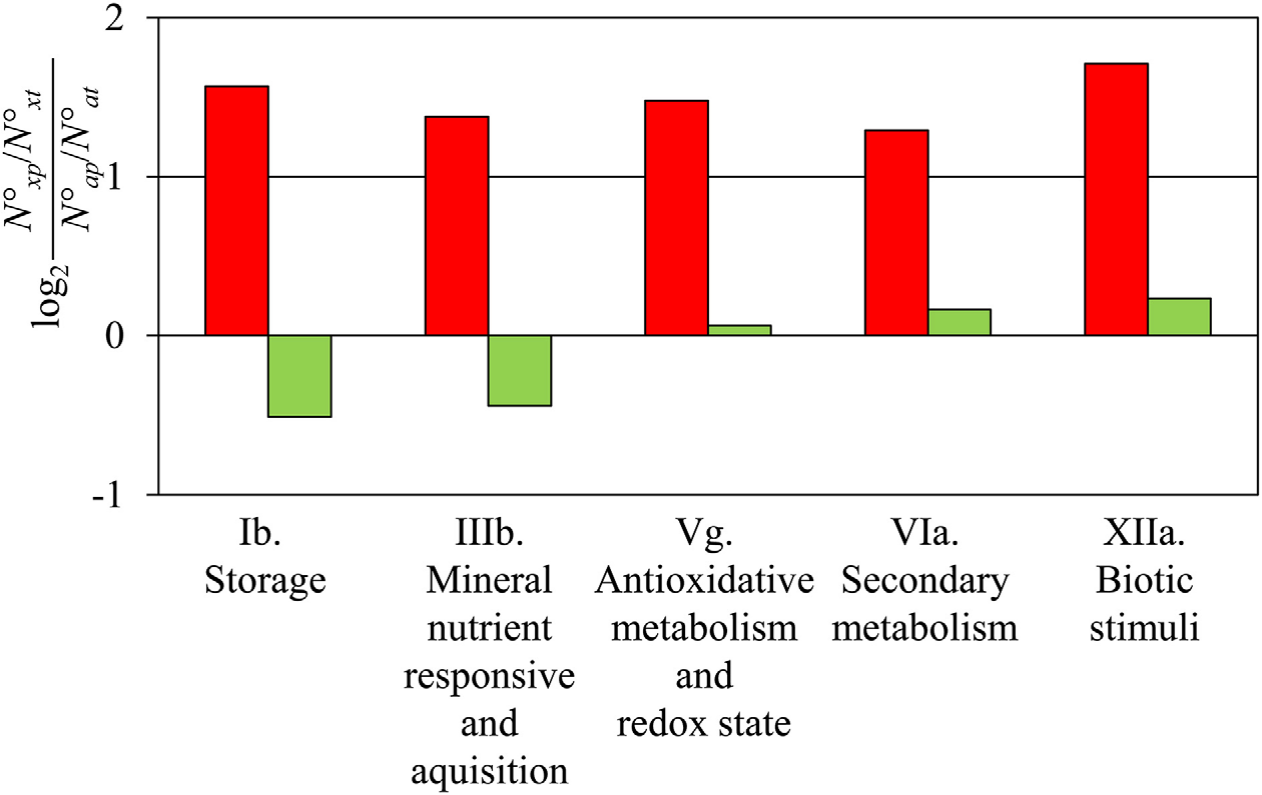
**Comparison of gene expression at 72 hpe vs. 24 hpe**. The five functional categories (Ib. Storage, IIIb. Mineral nutrient responsive and acquisition, Vg. Antioxidative metabolism and Redox state, VIa. Secondary metabolism, XIIa. Biotic stimuli, according to Table S1) showed a 2-fold higher number of up-regulated genes than expected by chance in the comparison 72 vs. 24 hpe (red columns). No category was detected with a significant 2-fold higher or lower number of down-regulated genes (green columns). *N*°_*xp*_, number of up- or down-regulated genes of a particular category, *N*°_*xt*_, number of all genes in the category; *N*°_*ap*_, number of all up- or down-regulated genes; *N*°_*at*_, number of all genes.

Comparing subsequent days of sampling, it became obvious that the largest shift between stem and root identity occurred between 24 and 72 hours post-excision (Table 1; Figure S1). These two stages were therefore directly compared (Table S1d). Detailed analysis of genes induced during AR formation from 24 to 72 h revealed that genes of the following five functional categories were over-represented in this group: storage, mineral responsive and acquisition, anti-oxidative metabolism and redox state, secondary metabolism, as well as biotic stimuli (Figure 1). The latter two categories include genes coding for laccases, polyphenol oxidases, and peroxidases (Table S1d) which may enhance the anti-oxidative capacity of the tissue. Also genes controlling the flavonoid pathway showed a particular shift in expression between 24 and 72 hpe (Table S1d) which may provide quantitative changes among particular pools. To find the category “storage” also overrepresented for induced genes was at first surprising. However, according to the three phase-model (Ahkami et al., 2009), the time point 72 hpe is situated in the recovery phase, which is characterized by the replenishment of resources. The resources might be stored also in vegetative storage proteins such as patatin-like proteins and class B acid phosphatases, whose encoding genes are highly expressed at this stage (Table S1d). No over- or under-representation of particular categories was detected when repressed genes were considered.

### Representation of phytohormone-related genes

**Figure 2:**
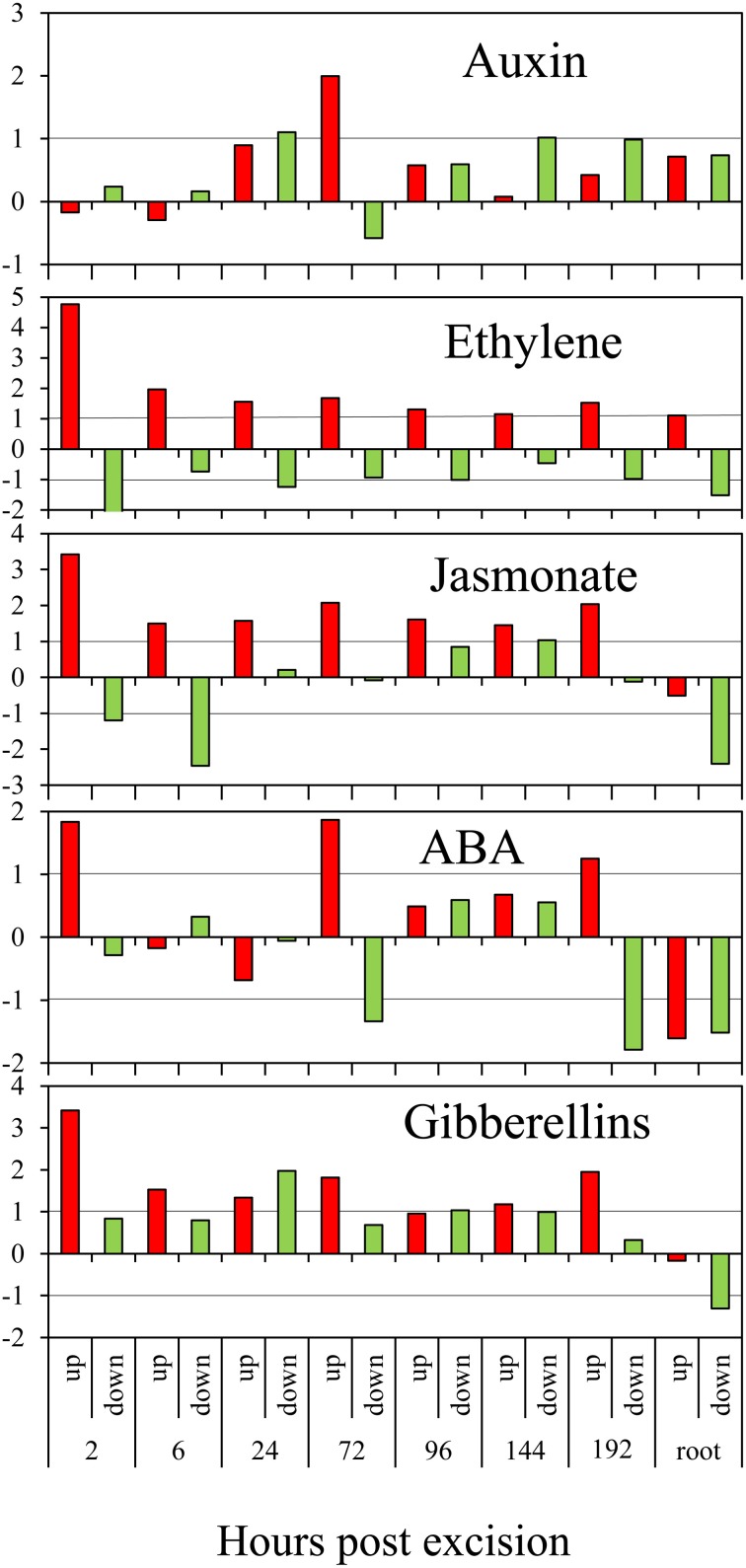
**Regulation of phytohormone-related genes during AR formation**. The ratios (*M*-values calculated as in Figure 1) of observed numbers vs. by chance expected numbers of up-regulated (red columns) or down-regulated (green columns) genes at different time points after excision or in roots compared to the stem base are shown on y-axis. ABA, abscisic acid.

The prominence of response of genes, which are related to the homeostasis and action of phytohormones was tested. Therefore, the numbers of statistically over- or under-represented category-specific genes showing induction or repression were determined for the different time points after excision (Figure 2). The numbers of genes in the categories “cytokinin,” “brassinosteroid,” and “salicylic acid” were too low for a reliable calculation. For the category “auxin,” repression of genes was slightly above the threshold of 2-fold overrepresentation at 24 hpe but was followed by a 4-fold overrepresentation of induced genes at 72 hpe. In contrast, the category “ethylene” showed the clearest but much more constant picture. Here, overrepresented induction was found at all the time points, with the most pronounced effect at 2 hpe. Similarly, jasmonate-related genes were continuously overrepresented in the pool of up-regulated genes, also showing the strongest distinction at 2 hpe.

Overrepresented up-regulation was further observed for the category “abscisic acid” at 2, 72, and 192 hpe (Figure 2). Interestingly, four of eight genes coding for 9-cis-epoxy-carotenoid dioxygenase (NCED) were repressed at 6 hpe (Table S1a). NCED catalyzes degradation of cis-neoxanthin and cis-violaxanthin to xanthoxin as direct precursor for ABA synthesis (Schwartz and Zeevaart, 2010) and may therefore contribute to a reduction of ABA to provide conditions favorable for AR induction. Subsequent induction of particular NECD-coding genes may be stress-induced and provide an increase in ABA level to adjust the tissue to stress conditions. Interestingly, genes coding for carotenoid cleavage dioxygenases (CCDs) which may also include NCEDs, were continuously down-regulated between 3 and 6-times during AR formation (Table S1a). Considering that in addition to ABA biosynthesis, CCDs are involved in strigolactone biosynthesis (Drummond et al., 2009), down-regulation of these enzymes may also contribute to a reduction of strigolactones in the rooting zone.

Additionally, genes in the category “gibberellin” showed an overrepresentation of induced genes at 2 hpe. Thereafter, they exhibited a more distinct pattern as both, induced and repressed genes, were overrepresented at different time points after excision (Figure 2). The responses of genes coding for different enzymes of GA metabolism do not provide a clear picture (Table S1a). Six genes encoding a recently identified GID1 gibberellin receptor (Sun et al., 2010) and a DELLA protein functioning as a GA response repressor were up-regulated without showing a clear phase dependency (Table S1a). However, particular up-regulation of one gene coding for a gibberellin-regulated GASA/GAST/Snakin family protein during the early period until 3 hpe (Seq_ID in supplemental tables: GO_drpoolB-CL4258Contig1) indicates functions in induction of AR. Induction of one petunia homolog of GAST1 (Ben-Nissan et al., 2004) between 72 and 192 hpe (Seq_ID in supplemental tables: cn3295) may indicate functions during AR formation, as already suggested for lateral root formation (Zimmermann et al., 2010).

We detected only very few genes of the cytokinin category regulated during AR formation (Table S1a). Interestingly, two of four genes coding for zeatin O-glucosyltransferase, catalyzing O-glucosylation of zeatin (Sakabibara, 2010), were highly induced during AR formation, one from 2 hpe onwards already. Considering that cytokinin *O*-glucosides are assumed to represent reversibly inactivated storage forms of cytokinins (Rodo et al., 2008), this response may contribute to the reduction of physiological active cytokinins in the rooting zone to stimulate AR induction. However, during the period between 2 and 24 hpe, a cytokinin response factor is up-regulated (Table S1a), which may indicate that cytokinin action is required during early induction of AR in petunia.

### Response of the auxin action machinery during adventitious root formation

Considering the recent findings that PAT and early accumulation of IAA are essential factors for excision-induced AR formation in petunia cuttings (Ahkami et al., 2013), we analyzed in detail the transcriptome of specific auxin-related genes of different functional categories. This revealed a complex response at the levels of auxin metabolism, transport, perception and down-stream signaling (Table 2, Table S1e).

**Table 2:**
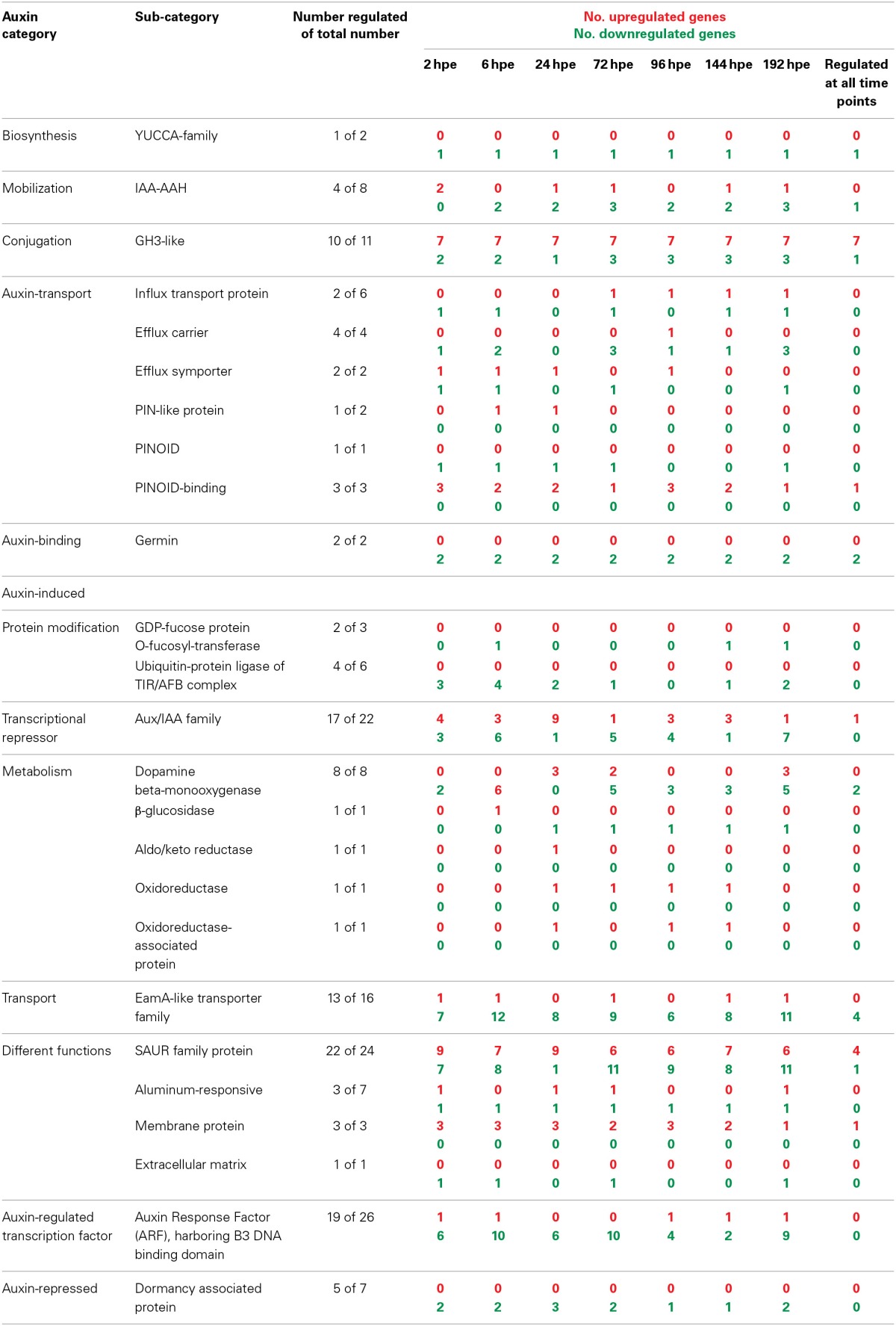
**Number of AR formation-regulated genes involved in auxin biosynthesis, signaling, or regulated by auxin**.

With regard to auxin biosynthesis, one gene encoding a flavinmonooxygenase of the YUCCA family was repressed throughout the rooting period from 2 hpe onwards. The expression of genes encoding two isoforms of IAA-amino acid hydrolases (IAA-AAH), which control the release of IAA from amino acid conjugates, was strongly induced at least 10-fold at 2 hpe. This was followed by a consistent down-regulation below initial expression values thereafter. It is important to note that the two IAA-AAH encoding genes were also strongly induced in leaves by wounding (Table S1a). This leads to the assumption that a wound response is involved in the induction of their expression in the stem base. Nevertheless, these changes in transcript accumulation may lead to local inputs of IAA. However, this is accompanied by the simultaneous but also prolonged induction of 7 out of 11 genes encoding proteins of the GH3 family, which potentially control auxin conjugation, whereas only few members of this gene family appeared to be down-regulated (Table 2). In four cases, associated transcript levels exhibited a first maximum at 2 hpe, declining at 6 hpe and started to increase again at 24 hpe (Table S1e). But two other GH3 genes (one of them, SEQ_ID in supplemental tables: GO_drpoolB-CL42Contig2, had a very high homology with GH3.3) showed an even stronger induction at 2 hpe and a further up-regulation until 6 hpe up to 100-fold.

Expression of diverse genes encoding components controlling auxin transport was also changed during AR formation (Table 2, Table S1e). Transcripts of two influx carriers were reduced until 6 hpe and of one of them also during later stages of AR formation. By contrast, the gene for one other influx protein was continuously induced from 24 hpe until 192 hpe. Expression of genes for individual efflux carriers responded differentially until 24 hpe showing both up- and down-regulation. However, genes for one PIN-like auxin transport protein and for one other auxin efflux hydrogen symporter were induced from 6 and 2 until 24 hpe, respectively, the latter reaching a 20-fold increase. During later stages of AR formation, efflux carriers genes were mostly down-regulated. One gene coding for the serine-threonine kinase PINOID, which catalyzes phosphorylation of PIN proteins, was down-regulated at most time points, whereas three genes for PINOID-binding proteins were up-regulated, one of them continuously. Transcription of two auxin-binding germin genes was continuously repressed.

With regard to the auxin perception machinery, genes encoding components of the TIR/AFB-complex and also of ARF proteins were repressed in most cases, beginning already at 2 hpe (Table 2, Table S1e). Individual ARF genes were down-regulated most frequently during the early phase of AR formation until 72 hpe and at the very late stage of AR formation. However, among the transcriptional repressor family of Aux/IAA-like proteins 17 out of 22 putative genes were regulated and showed the most phase-specific regulation of gene expression. Here, the strongest shift occurred during the period from 6 hpe until 72 hpe (Table 2, Table S1e). Between 6 and 24 hpe, the number of induced genes increased by six whereas the number of repressed genes decreased by five. Only one of nine differently induced genes remained at this state until 72 hpe, whereas five other genes became repressed. Considering other families of auxin-induced genes, those encoding SAUR-like proteins were most responsive (22 of 24 genes) showing both up- and down-regulation during AR formation. Similar to the Aux/IAA family, genes coding for SAUR-like proteins showed the strongest shift in expression between 6 and 72 hpe (Table 2, Table S1e).

### Ethylene in adventitious root formation

**Figure 3:**
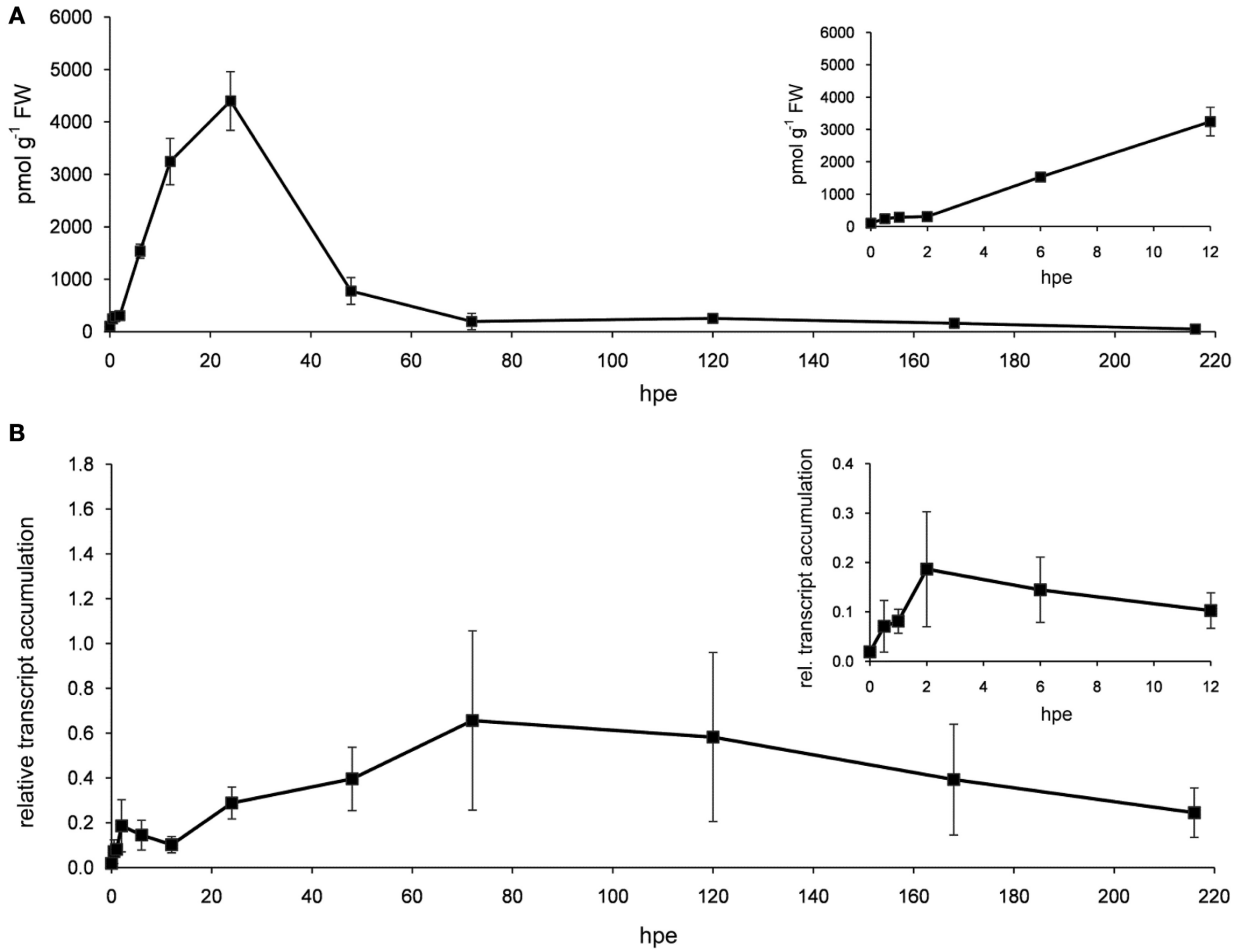
**ACC and *ACO* transcript levels**. Stem bases of cutting were harvested at different time points after excision and used for measuring the accumulation of ACC **(A)** and transcripts of *PhACO1* **(B)**. Relative transcript accumulation in **(B)** was determined using *PhCyRiPro* as constitutively expressed gene. Shown are means and standard deviations of three biological replicates. Insets show details of early time points.

Detailed analysis of ET-related genes revealed that mostly three functional sub-categories contributed to the prominent and constant induction of this category (Table 3). Genes encoding aminocyclopropane-1-carboxylic acid (ACC) synthase (ACS) and ACC oxidase (ACO) were highly induced at transcriptional level after excision of cuttings from 2 hpe onwards (Table S1a). Their high transcript levels were maintained throughout all phases of AR formation. Four genes encoding ACS were continuously up-regulated. From 6 hpe onwards, between six and eight of ACS-encoding genes were induced (Table 3) mostly showing similar expression ratios for the different time points while three genes exhibited maximum induction at 24 hpe (Table S1f). Regarding ACO, highest number of induced genes was found at 96 hpe, while the maximum up-regulation per gene varied between the time points depending on the particular gene. Increase in ET biosynthesis could be confirmed by determination of the levels of ACC and transcripts of *PhACO1* (cn1774). ACC accumulated to highest levels after 24 h and declined later again to a basic level at 72 h (Figure 3A). RNA level of *PhACO1* was sharply increased at 2 hpe, then decreased up to 12 hpe and slowly increased again until 72 hpe (Figure 3B). This pattern was not only visible for cn1774, but also for some other sequence identifiers for *ACO* genes in the array (Table S1f).

**Table 3:**
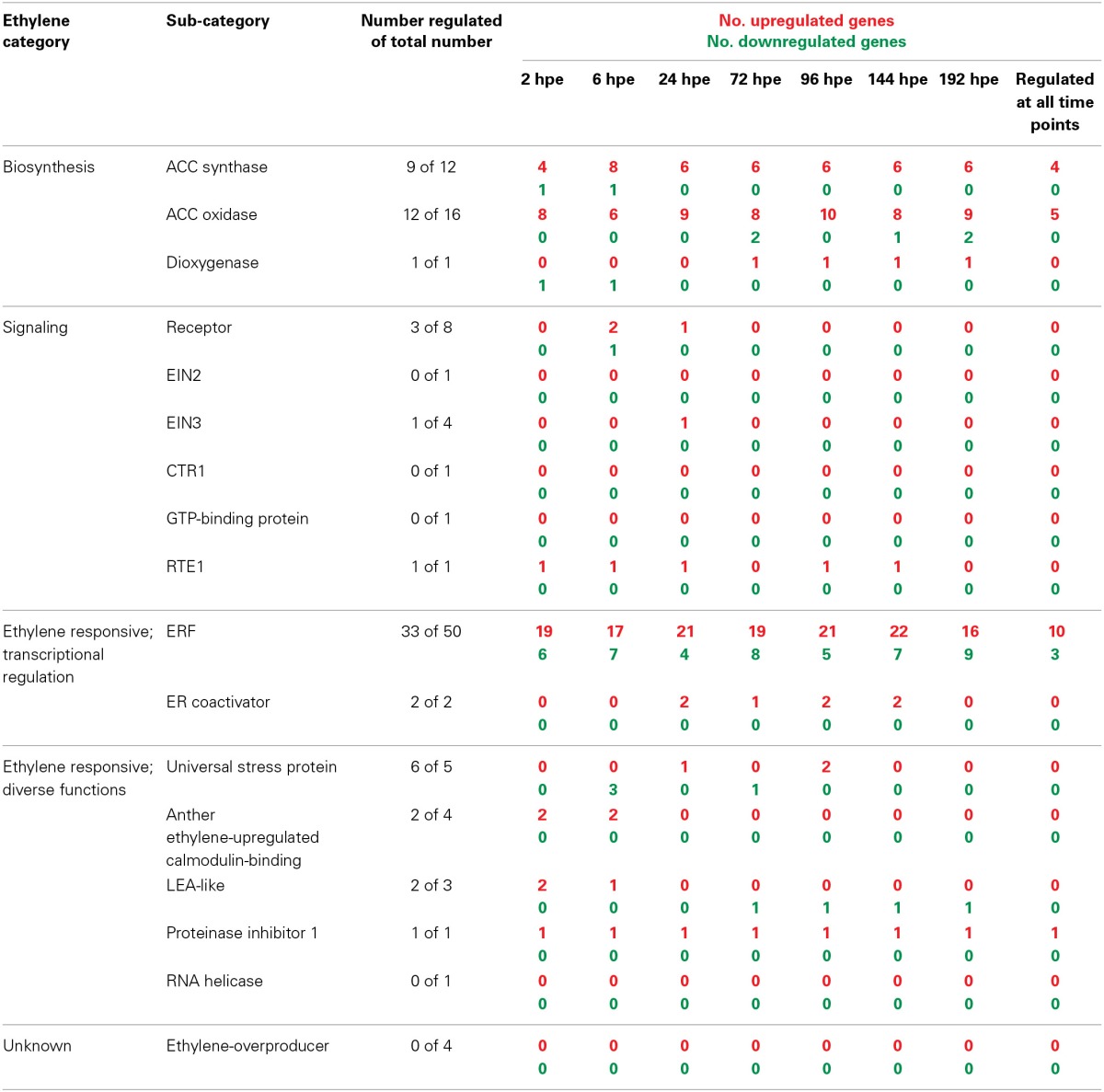
**Number of AR formation-regulated genes involved in ethylene biosynthesis, signaling, or regulated by ethylene**.

Considering components of the ET perception and signaling pathway, only 2 of 8 genes coding for ET receptors were induced 2-fold at 6 and 24 hpe, while another gene was repressed by two times at 6 hpe. The expression of one gene encoding EIN3, a positive regulator of ET signaling downstream of the ET receptors (Gazzarrini and McCourt, 2003), was doubled at 24 hpe, whereas RTE1, a negative regulator of ET signaling (Resnick et al., 2006), was induced up to 6-fold at almost all time points. However, with regard to ET signal transduction, the most pronounced response to cutting excision and AR formation was observed for genes encoding ET responsive transcription factors (ERFs). From 2 hpe until 192 hpe, between 17 and 22 out of 50 identified ERF genes were induced, 10 of them constantly (Table 3). Thirteen of the ERFs induced during this period were also induced in leaves by wounding. During the same period, between four and nine ERF genes were repressed, three of them constantly. One of the repressed ERFs was also repressed by wounding. From the 33 regulated ERF genes 24 showed exclusively induction, whereas seven showed repression only. Further responses of ET regulated genes included induction of two coding for ER co-activators between 24 and 144 hpe, very short-termed inductions or repressions of genes coding for particular stress proteins, induction of two anther ET-calmodulin binding protein genes at 2 hpe and a constant induction of one gene encoding the proteinase inhibitor 1. Interestingly, LEA-like proteins showed a phase-specific expression pattern (Table 3). Induction between 2 and 6 hpe was followed by repression from 72 hpe onwards, while both responses involved the same gene (Table S1f).

**Figure 4:**
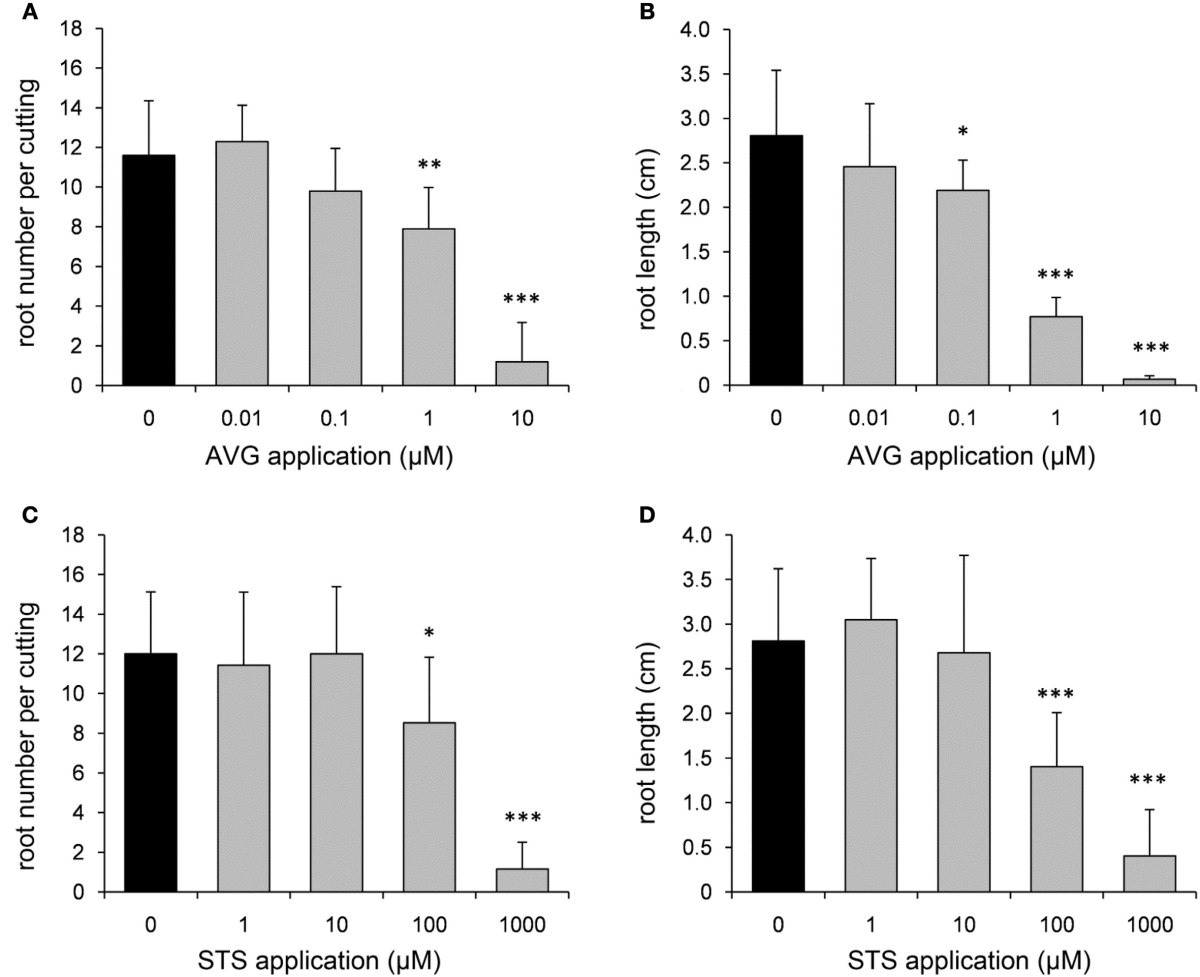
**Effects of aminoethoxyvinylglycine (AVG) and silver thiosulfate (STS) on number and length of ARs**. In order to inhibit ethylene biosynthesis and ethylene perception, different concentrations of AVG **(A,B)** or STS **(C,D)** were applied to de-rooted seedlings. Number of roots **(A,C)** and average root length **(B,D)** were assessed after 14 days. One out of three independent experiments showing similar results is presented. Significant differences to mock-treated cuttings (0 μM) are indicated by asterisks (*n* = 10; ^*^P < 0.05, ^**^P < 0.01, ^***^P < 0.001 according to Student's *t*-test).

**Figure 5:**
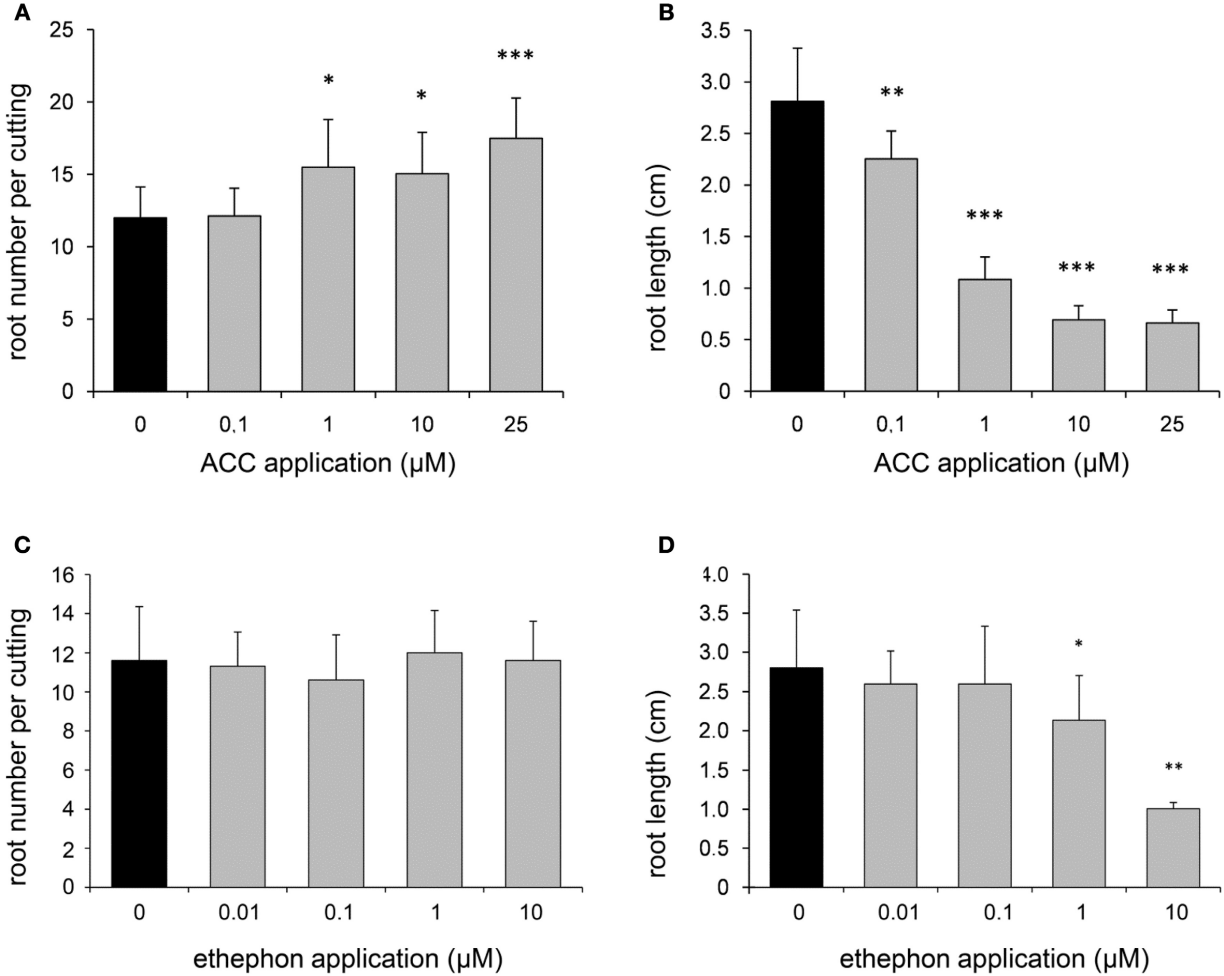
**Effects of aminocyclopropane carboxylate (ACC) and ethephon on number and length of ARs**. In order to simulate ethylene overproduction, different concentrations of ACC **(A,B)** or of ethephon **(C,D)** were applied to de-rooted seedlings. Number of roots **(A,C)** and average root length **(B,D)** were assessed after 14 days. One out of three independent experiments showing similar results is presented. Significant differences to mock-treated cuttings (0 μM) are indicated by asterisks (*n* = 10; ^*^P < 0.05, ^**^P < 0.01, ^***^P < 0.001 according to Student's *t*-test).

In order to further elucidate the role of ET in AR formation in petunia, de-rooted seedlings were treated with either AVG as an inhibitor for ET biosynthesis or with STS as inhibitor of ET perception. Both compounds clearly reduced the number of ARs and the average root length with increasing concentrations starting between 0.1 and 1 μM for AVG and above 100 μM for STS (Figure 4). To study the effects of increased ET levels, the biosynthesis precursor ACC or the ET-generating compound ethephon were applied. ACC increased the number of roots per cutting at a concentration of 1 μM or higher, but resulted in reduced average root length with increasing concentrations (Figure 5A). Ethephon application had no significant effect on root numbers, whereas root length was diminished at concentrations higher than 1 μM (Figure 5B). The results clearly demonstrate the dependency of AR formation in petunia on ET biosynthesis and ET signal perception, while the reduced root length in response to high concentrations of ACC and of ethephon reflect an inhibitory role of high ET levels in root elongation.

## Discussion

Plants are organisms with a very high capacity for dedifferentiation of tissues and cells followed by new determination of organ identities and differentiation and growth of these organs. It can be assumed that these processes are accompanied by massive changes in the expression of numerous genes. It is therefore reasonable to analyze expression patterns during such a process as comprehensively as possible. This can be best achieved by technologies like screening of arrays followed by analysis of the expression values obtained for the sequences on these arrays and accompanied by annotation of the sequences and classification of the putative functions. However, up to date microarray technology has only rarely been applied to investigate AR formation. Brinker et al. (2004) analyzed the expression of ca. 2200 sequences during AR formation in cuttings of *Pinus contorta*, which, however, was induced by external application of auxin. Abu-Abied et al. (2012) used a microarray for comparison of juvenile and mature cuttings of *Eucalyptus grandis* before root induction, but did not monitor the dynamic of AR formation. In a recent study, we applied a microarray to analyze the expression of about 25,000 unique, non-redundant annotated sequences during spontaneous AR formation in *P. hybrida* cuttings (Ahkami et al., 2014). In that study, however, the developmental process was subdivided into three metabolic phases according to Ahkami et al. (2009) and the respective dates (6, 72, and 192 hpe) were analyzed focusing on the plant primary metabolism.

In the present evaluation of the data generated by Ahkami et al. (2014), we considered AR formation as a continuous process where in the SB of the cutting particular cells may be de-differentiated first or directly start from the non-differentiated state to be newly determined as root meristems. These cells are then developing into root primordia and the complete body of the root leading to an increasing number of cells with root identity. Considering these switches in identity of the stem tissue, our objective was to track the involvement of plant hormone-related genes. Genes expressed in SB at the time of excision and genes expressed in fully developed roots were used to distinguish between stem and root identity. Evaluating differential expression of these genes, the strongest shift toward root identity was observed between 24 and 72 hpe (Figure S1), when also first meristematic cells of the developing root meristem appear (Ahkami et al., 2009, 2014). This shows that particular root functions are already exerted when first meristems for AR develop.

### Changes in anti-oxidative and flavonoid metabolism may modify auxin homeostasis

Interestingly, the five functional categories showing the most prominent induction during this period (Figure 1) include genes which on the one hand may have functions in the protection of the tissue against stress-induced reactive oxygen species and on the other hand may influence plant hormone homeostasis and signaling. The first aspect may particularly apply to the induced genes putatively encoding laccases, polyphenol oxidases and peroxidases (Table S1d). Interestingly, high antioxidant enzyme activities have been already observed in calli during *in vitro* organogenesis (Vatankhah et al., 2010). Furthermore, root growth is inhibited by the addition of H_2_O_2_, which affects the expression of cell cycle-related genes, and this inhibition can be released by overexpressing a peroxidase-encoding gene in *Arabidopsis* (Tsukagoshi, 2012). Another role of these enzymes important in AR formation could be their involvement in lignin polymerization, which might have a wound-healing function, but studies of lignin biosynthesis and/or deposition are necessary to confirm such a hypothesis. Considering the catalytic activity on IAA (da Costa et al., 2013), the pronounced increase in the expression of peroxidases (Table S1a) may additionally contribute to the decline of IAA after 24 hpe and subsequent maintenance of low levels, which was observed under same experimental conditions (Ahkami et al., 2013) as applied in the experiments generating the data analyzed in the present study. Also the shift in the expression of genes coding for enzymes of the flavonoid pathway can be expected to modify auxin homeostasis. Flavonoids modify auxin transport particularly by interaction with efflux carriers (Peer and Murphy, 2007; Santelia et al., 2008) while different flavonoids show different such activities (Buer et al., 2013). Furthermore, flavonoids can buffer auxin-induced ROS accumulations and interfere with ROS-dependent IAA catabolism to 2-oxindole-3-acid acid, which is considered an important process of auxin signal attenuation (Peer et al., 2013).

### Excision of cuttings causes a fine-tuning of the auxin transport system, down-regulation of auxin level and sensitivity, and phase-specific changes in the Aux/IAA-ARF machinery

Complementing our recent finding that induction of AR formation in petunia cuttings is highly dependent on PAT and on a transient IAA-peak arising at 24 hpe (Ahkami et al., 2013), the present evaluation of transcriptome data provides the first view on possible major control points of auxin in relation to excision-induced AR formation at the levels of metabolism, transport, signal perception and downstream signaling.

In accordance to the strong dependency of IAA accumulation on PAT (Ahkami et al., 2013), the trancriptome data does not indicate up-regulation of genes involved in local auxin biosynthesis (Table 2). The strong but very time-restricted induction of two IAA-AAH genes at 2 hpe indicates that the IAA accumulation until 2 hpe (Ahkami et al., 2013) might be at least partially the outcome of conjugate hydrolysis. The later repression of same genes may contribute to the observed reduction of the IAA pool. Hydrolysis of IAA-conjugates is considered as important event controlling auxin homeostasis (Ljung et al., 2002). Additionally, the role of early hydrolysis is supported by the fact that IAA accumulation at 2 hpe in the SB is insensitive to application of a PAT-blocker (Ahkami et al., 2013). Induction of genes encoding GH3-like proteins accompanied increasing IAA levels and mirrors results of northern blot analyses using the petunia *GH3* gene CV296522 under same experimental conditions (Ahkami et al., 2013). One major function of GH3 proteins is their activity as IAA-amidosynthetases, which are important for maintaining auxin homeostasis via conjugation of IAA to amino acids (Staswick et al., 2005). Specific *GH3* genes may have other particular functions during the induction phase of AR formation. Recently, Gutierrez et al. (2012) showed that three auxin-inducible *GH3* genes including one *GH3.3* were required for positive regulation of light-induced AR formation in hypocotyls of intact *Arabidopsis* seedlings, possibly via conjugation of inhibitory JA. The prominent induction of jasmonate-related genes (Figure 2, Table S1a) points to an involvement of JA in AR formation also in cuttings. However, in petunia cuttings JA shows an early accumulation peaking at 0.5 h followed by a very fast decline to initial level so that early JA accumulation was suggested to contribute to AR formation via sink establishment in the rooting zone (Ahkami et al., 2009). Further analysis with high time resolution at levels of metabolites and gene expression of candidate genes in combination with pulse applications at distinct time points is necessary to elucidate the role of JA during the different phases of AR formation in cuttings.

The present evaluation of transcriptome data does not indicate a general stimulation of the auxin transport machinery after excision, but rather points to a fine-tuning of distinct events. However, it has to be considered here, that even when auxin biosynthesis, metabolism and transport would remain unchanged in the stem base, auxin should accumulate after excision of cuttings, because separation from the basal part of the plant should interrupt further basipetal transport from the stem base. Microarray analyses of gene expression during auxin-induced AR formation in cuttings of *P. contorta* revealed repression of an ATP-binding cassette (ABC) transporter and of an AUX1-like gene at day 3 after excision of cuttings and auxin application (Brinker et al., 2004). Sukumar et al. (2013) provided evidence that localized induction of the ABC B19 auxin transporter contributes to AR formation in *Arabidopsis* hypocotyls in response to excision of roots. The characterization of an auxin influx carrier in cuttings of carnation during cold dark storage showed its generally higher expression in the rooting zone (basal internode) compared to the upper node (Oliveros-Valenzuela et al., 2008). In the same plant species, two genes encoding another putative auxin influx facilitator and a putative PIN-like efflux carrier were up-regulated 15 hours post-excision of cuttings, which was stimulated by auxin application (Agullo-Anton et al., 2014). PIN1 has a particular role for PAT in *Arabidopsis* (Gälweiler et al., 1998) and for spontaneous AR formation in rice plants (Xu et al., 2005). Thus, the observed early up-regulation of genes for the PIN-like protein and for another auxin-hydrogen symporter (Table 2, Table S1e) may have important functions for early auxin accumulation and induction of AR in petunia cuttings. This may particularly apply to the situation at 24 hpe, when no simultaneous repression of other efflux carriers is observed (Table S1e) and the IAA level is most sensitive to blocking of PAT (Ahkami et al., 2013). By contrast, influx carrier genes were down-regulated during the induction phase, whereas one particular influx transport protein gene was continuously up-regulated thereafter (Table S1e). This finding suggests an important role of auxin influx during the formation of new root meristems and subsequent differentiation. Such a role of auxin influx carriers would stay in line with the proposed functions of AUX1 to promote the acropetal, post-phloem movement of auxin to the *Arabidopsis* root apex (Swarup et al., 2001) and of AUX/LAX controlled auxin influx as important factor controlling embryonic root and lateral root development (Peer et al., 2011).

For direction of auxin flow, PIN localization between basal (rootwards) and apical (shootwards) membrane domains is essential. There is strong indication in literature that high activity of the protein kinase PINOID targets PINs to the apical plasma membrane probably via PIN phosphorylation while the phosphatase complex PP2A acts antagonistically (Friml et al., 2004; Michniewicz et al., 2007; Fozard et al., 2013). Considering these functions, the observed down-regulation of one PINOD gene until 72 hpe (Table 2) may contribute to basal localization of PINs and thus contribute to basipetal auxin flux in the rooting zone. However, the consequence of the observed up-regulation of genes coding for PINOID-binding proteins for the auxin flux is unclear. PINOID-binding proteins seem to modify PINOID activity, while the direction of influence obviously depends on the particular protein and on calcium levels (Benjamins et al., 2003). Nevertheless, the expression data points toward a fine-tuning of PIN localization during AR formation. The changing IAA levels (Ahkami et al., 2013) might be involved in these responses, since feedback loops between auxin levels and expression and localization of PINs were shown (Benjamins and Scheres, 2008).

The knowledge of different steps of auxin perception and signaling during AR formation is only fragmentary (Pop et al., 2011). The present evaluation of transcriptome data clearly indicates that shortly after excision of cuttings up to 72 hpe many genes putatively encoding ubiquitin-protein ligases of the TIR/AFB complex and ARFs were down-regulated (Table 2). In contrast, their up-regulation was only rarely observed. Considering the positive roles of these components in auxin perception (see Introduction Section), the results reflect a transcriptomic response toward overall reduction of auxin sensitivity in the SB after excision. Also these responses may be based on a negative feedback to the early rise in IAA level (Benjamins and Scheres, 2008; Ahkami et al., 2013).

Because certain ARFs may act as repressors of AR formation (Gutierrez et al., 2012) down-regulation of certain ARFs particularly during the period until 72 hpe may contribute to the induction of AR formation in petunia. Interestingly, expression of ARFs and Aux/IAAs is generally considered as important “auxin codes” for the programming of developmental phases (Teale et al., 2006). The observed strong temporal variation in the expression of genes of the Aux/IAA-family (Table 2) supports the view that these are important selective controllers for particular auxin response pathways in relation to AR formation in petunia. Furthermore, the strong shift between 6 and 72 hpe in expressions of Aux/IAAs supports their important role for the completion of the induction and transition into the subsequent root formation phases. Since the expression of Aux/IAA proteins is highly sensitive to auxin (Benjamins and Scheres, 2008) the observed switches in expression particularly during the induction phase can be expected to be controlled by the changes in IAA concentration (Ahkami et al., 2013). Furthermore, considering reported responses of expression of Aux/IAA also to other plant hormones (Brenner et al., 2005; Song et al., 2009; Cakir et al., 2013), certain Aux/IAA may also provide linkages for hormonal crosstalk during AR formation. Auxin-binding protein 1 (ABP1), which belongs to the family of germin-like proteins (Carter et al., 1998; Membre et al., 2000), is considered as early sensor of apoplastic auxin concentrations regulating auxin transport and early, fast, transcriptional-independent, membrane and cytosolic responses (Scherer, 2011). It obviously acts further as negative regulator in the SCF TIR1/AFB pathway (Tromas et al., 2013). Interestingly, two genes coding for auxin-binding germins were continuously repressed during AR formation. Further characterization of these two genes is necessary to elucidate whether they have auxin reception functions similar to those of ABP1. The observation that almost all identified genes coding for SAUR-like proteins were up- or down-regulated (Table 2) suggests important roles of specific SAURs in AR formation of petunia. Furthermore, the strong modulation of the expression between 6 and 72 hpe suggest that specific SAUR-like proteins have particular functions in the induction and early differentiation of ARs. However, even though different functions have been linked to SAURs (Park et al., 2007; Kant et al., 2009) their function in AR formation is completely unknown.

### Stimulation of ethylene synthesis and ERF-mediated signaling during AR formation

The present evaluation of transcriptome data indicates a strong stimulation of ET biosynthesis at transcription level, starting already at 2 hpe. Furthermore, ET biosynthesis at the stages of ACC synthesis and ACC oxidation responds in a similar manner showing induction during all phases of AR formation while the time points of maximum induction varied between individual genes (Table 3, Figure 3B, Table S1f). These transcript accumulations suggest different but overlapping principles of stimulation. At first, ET biosynthesis is at both levels of the pathway sensitive to a diverse set of abiotic stresses with an outstanding role of wound stress (Druege, 2006, and references therein). Particularly in vegetative tissues, production of wound ET often has an explosive but transient character leading to a burst of ET evolution within a few hours and a rapid and strong decline to low levels thereafter (Einset, 1996; Shiu et al., 1998; Tatsuki and Mori, 1999). Induction of both ACS and ACO has repeatedly shown to be involved and ACC generated by ACS may contribute to the wound-induced induction of ACO (Nie et al., 2002). The injury of excised cuttings might contribute to the early induction of ACS and ACO. This is supported by the fact that three genes coding for ACS and five genes encoding ACO showed also induction in leaves within 2 h after wounding (Table S1a). Unfortunately, detection of ET via GC and flame ionization according to Kadner and Druege (2004) was not sensitive enough to monitor ET evolution from the cuttings base of petunia (data not shown). Therefore, we monitored ACC as immediate precursor of ET. The strong but transient rise of ACC peaking at 24 hpe (Figure 3A) further confirms the excision-induced stimulation of the ET biosynthetic pathway in the rooting zone of petunia. However, the difference between the peak of ACC accumulation and the continuous induction of genes coding for ACS and for ACO (Table 3, Table S1f) clearly demonstrates the importance of post-transcriptional regulation of ET biosynthesis in petunia. Because ET synthesis may be limited by ACO and in consequence may even show a reverse trend to ACC levels (Liu et al., 1990), ACC levels do not provide information about the strength of the ET signal. Advanced methods such as photo-acoustic detection should be involved in future studies to monitor ET emission of cutting tissues.

In addition to wounding, the isolation of cuttings from the whole plant includes cutting off from the water flow and from all other root-sourced influxes of signals and resources. Considering that a high photosynthetic rate is maintained in the young petunia shoots from the beginning after excision from the donor plant (Klopotek et al., 2012) but water uptake is usually reduced in cuttings (Loach, 1988), a temporary water deficit can be expected in the cutting tissues which may have stimulated ET biosynthesis (Druege, 2006). Because also the influx of root-sourced nutrients is interrupted in the cutting, the found stimulation of ethylene biosynthesis may be partially the consequence of nutrient deficiency. Regarding on the one hand that mineral transporters including Fe-transporters are induced at 72 hpe in the rooting zone of petunia cuttings possibly indicating nutrient deficiency (Ahkami et al., 2014) and on the other hand that in roots of *Arabidopsis* Fe deficiency up-regulates genes coding for ACS and oxidases (Garcia et al., 2010), isolation-induced Fe deficiency may have contributed to the observed later induction of ET biosynthesis regulating genes.

Considering the accumulation of IAA between 2 and 24 hpe in the rooting zone under same experimental conditions (Ahkami et al., 2013) and the response of genes of the auxin category (Table 2), the induced expression of genes limiting ET biosynthesis may be partially regulated by auxin action. There exists extensive crosstalk between auxin and ET at the levels of metabolism, transport and signaling (Negi et al., 2010; Muday et al., 2012; Pacheco-Villalobos et al., 2013). While ET has been shown to stimulate synthesis of IAA in roots (Ruzicka et al., 2007; Swarup et al., 2007), application of auxin including IAA enhanced transcript levels of ACS and ACO in plants (Peck and Kende, 1995; Yun et al., 2009; Wilmowicz et al., 2013).

The inhibition of AR formation by AVG (Figure 4A), an inhibitor of ACS activity (Yang and Hoffman, 1984) demonstrates the importance of ET biosynthesis and particularly of the ACC pool for AR formation in petunia cuttings. Complementary to this response, application of ACC to de-rooted seedlings showed a stimulation of root number (Figure 4A). Contrasting to ACC, ethephon, a substance which directly releases ET, had no effect on root number even though a broad range of concentrations was tested (Figure 4B). This may indicate a very narrow range of ET concentrations for induction of AR formation, which may have not been attained. In this context, it has to be considered that application of ACC enhances ET biosynthesis within the physiological limit of the plant. Because ACC has to be converted by the endogenous ACO, the resulting magnitude and place of ET enhancement is under control of the plant. This is not the case with ethephon, which releases ET to be transported everywhere. However, the reduction of root length by both ACC and ethephon at high concentrations stays in line with the repeatedly observed inhibition of root elongation by high ET concentrations, which has been observed in both primary and ARs (Krishnamoorthy, 1970; Riov and Yang, 1989; Negi et al., 2008). According to a current concept, ET does not reduce root growth directly but rather stimulates biosynthesis and transport of auxin into the elongation zone, where it inhibits root elongation (Rahman et al., 2001; Stepanova et al., 2005, 2007; Ruzicka et al., 2007; Muday et al., 2012).

Considering that ET receptors function as negative regulators of ET response when ET is not bound (Cancel and Larsen, 2002; Alonso and Stepanova, 2004), the weak induction of two of 8 genes putatively encoding ET receptors at 6 hpe (Table 3) may provide a temporary slight reduction in ET sensitivity. This may be the response to stimulation of ET biosynthesis (Yau et al., 2004; Druege, 2006). Cuttings of two transgenic lines of petunia “Mitchell” which either constitutively expressed the *Arabidopsis* mutant ET receptor *etr1-1* or showed a reduced expression of *PhEIN2*, the petunia homolog of *Arabidopsis EIN2* gene coding for a positive ET regulator, were strongly inhibited in AR formation as reflected by reduced number, length and dry matter of ARs (Clark et al., 1999; Shibuya et al., 2004). According to these findings, the strong reduction of AR formation found in the present study in response to treatment with STS which interferes with the ethylene-receptor binding (Sisler et al., 2006) clearly demonstrates the dependence of AR formation in petunia cuttings on ET perception.

The broad and over the whole period of AR formation induced transcription of most ERF-encoding genes (Table 3, Table S1f) and the simultaneous repression of other ERF genes clearly demonstrate the importance of ET signaling during AR formation in petunia cuttings. ERFs are transcription factors regulating ET-responsive genes, while their expression responds to ET and to a diverse array of extracellular stimuli including abiotic stress (Ohme-Takagi et al., 2000). They are considered to affect developmental processes particularly in the frame of environmental stimuli or hormones (Licausi et al., 2013). Considering the plenty of regulated ERFs (Table 3) and the multiplying function of these transcription factors in regulation of other genes (Mizoi et al., 2012), the results reflect a strong impact of ET on gene expression during the whole process of AR formation. Interestingly, it was shown that expression of a transcription factor of the AP2/ERF family in *Populus* controlled the intensity of AR formation (Trupiano et al., 2013). This effect was even enhanced by application of auxins and significant metabolic changes in the shoot suggested not a specific control of root development but rather a broader regulatory role of the gene. According to such a role, the analysis of expression of ERF genes in the present evaluation of transcriptome data does not mark certain time points of shifting activities neither at the level of gene number (Table 3) nor at the level of activities per gene (Table S1f) during the different phases of AR formation. Taken together, the whole expression data suggests that, unlike the phase-specific pattern of some auxin-related genes, transcription of genes involved in ET biosynthesis and response pathways is important to stimulate AR development but not to regulate the process *per se* (Table S2).

### Auxin as major regulator

**Figure 6:**
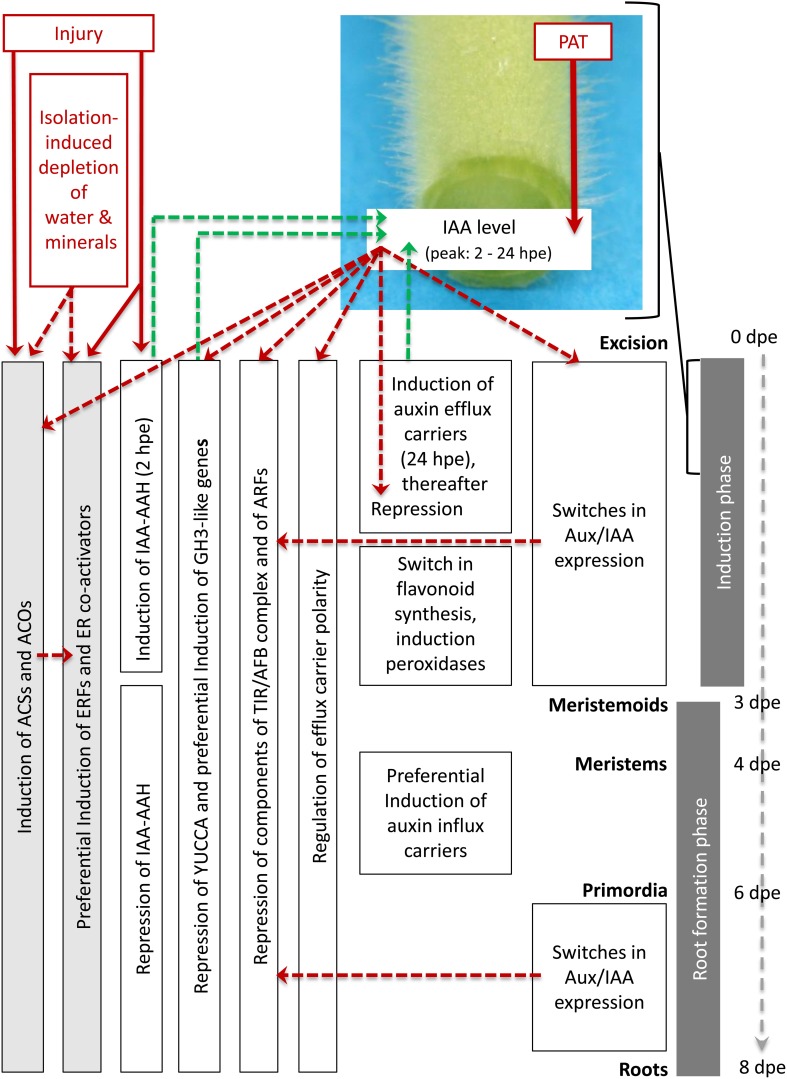
**Postulated model of regulation of ethylene and auxin biosynthesis, of auxin transport and of ethylene and auxin signal perception at transcriptome level during AR formation in petunia cuttings**. Red lettering marks important stimulating factors. Red arrows indicate the evident influence of PAT on IAA accumulation based on the results of Ahkami et al. (2013) and of injury on expression of ACSs, ACOs, ERFs, and IAA-AAH based on the present study. Red dashed arrows indicate hypothetically involved controls of gene expression based on the literature. Green dashed arrows indicate hypothetic links from gene expression to IAA levels based on the literature. For further explanation, see the text.

By contrast to ET, the transcriptional control of auxin action appears as major controlling process to induce and initiate the entrance into the particular phases of AR formation in petunia. Based on the phase-specific responses of the different sub-categories of auxin- and ET-related genes which are summarized in Table S2, and the results obtained by Ahkami et al. (2013) a model of transcriptional regulation of both plant hormones during AR formation is proposed in Figure 6.

Immediately after excision of cuttings, injury induces genes coding for IAA-AAH, ACS and ACO. The wounding and stimulated ET biosynthesis provokes induction of ERFs. Later on, water deficit and mineral deficiency in the cutting and auxin signaling contribute to the ongoing high expression of ACSs, ACOs, and ERFs. Shortly after excision, the current PAT leads to the accumulation of IAA in the rooting zone because the basipetal drain is cut off. This is supported by the activity of the short-termed induced IAA-AAH catalyzing the release of IAA and by preferential induction of particular efflux carriers such as PIN. From the beginning, repression of YUCCA genes and the auxin-induced up-regulation of GH3-like genes provide a subsequent decline of IAA, which is further supported by induction of peroxidase genes at 3 days post-excision (dpe) providing auxin oxidation. A switch in flavonoid metabolism may additionally reduce auxin transport and attenuate auxin signaling. Starting also immediately after excision, auxin-induced and Aux/IAA-mediated repression of components of the TIR/AFB complex and of ARFs provide a reduction in auxin sensitivity. These processes contribute to re-establishment of auxin homeostasis at the initially low IAA levels and to provide subsequent differentiation of root primordia from 3 dpe onwards. Auxin-induced switches in expression of the transcriptional repressors Aux/IAA provide auxin-driven induction of AR and the entrance into the early cell proliferation processes as well as the later differentiation of root primordia to fully developed roots, which occurs at low IAA levels. Later differentiation of root primordia occurring at low IAA levels involves preferential induction of auxin efflux carriers. Considering the intensive auxin-ET crosstalk in root development (Muday et al., 2012), the strong dependency of AR formation on ET biosynthesis and perception (Figures 4, 5) some of auxin-regulated processes should be mediated by ET action, while the Aux/IAA proteins may provide crossroads for both hormones (Chaabouni et al., 2009) and also to other hormones.

## Conclusion

AR formation is a process where continuously cells in the SB are reprogrammed to develop meristems for primordia and subsequent root development. On the molecular level it is characterized by an increased expression of root-specific and a decreased expression of stem-specific cells. This is, however, not a steady process, but involves an initial sudden induction of a large number of genes and a second quantitative rise at the appearance of the first meristematic cells. Molecular data indicate that this phase is characterized by the production of protective factors (antioxidants, secondary metabolites), which also may have functions in plant hormone homeostasis. Comparing the expression data of genes in the phytohormone categories supports the scenario that ET plays an important role in stimulating the process of AR formation through the different phases. The general dependency of AR formation in petunia on ethylene biosynthesis and reception has been confirmed here. In contrast, the regulation of genes involved in auxin-related processes is highly complex and appears to navigate through the different phases of AR formation. Cutting excision can be considered as a kind of “accident” to the shoot, where the basipetal drain of auxin is interrupted leading to an overflow of this phytohormone in the stem base, which induces AR formation. Using feedback loops to auxin transport and to auxin signaling, auxin appears to stimulate a shift of the transcriptome to (a) provide a buffering against the auxin overflow for re-establishment of auxin homeostasis and (b) adjust auxin signaling for subsequent initiation, differentiation and growth of new roots. This obviously involves a strong regulation of Aux/IAA proteins, which may also provide nodes for linkage to other plant hormones. The expression data shows regulation also of other transcription factors and other genes, which may be linked to auxin (Table S1a). However, these will be analyzed in further studies.

Based on the magnitude and the phase-specificity of induction or repression of transcription, candidate genes of the different functional categories were selected and listed in Table 4. Functional analysis of these genes by generating transgenic lines through sense or anti-sense approaches and by use of reporter constructs will allow to determine the roles in the different phases of AR formation and to assign the different functional activities to particular tissues or cells.

**Table 4:** **Candidate genes of different categories for auxin- and ethylene-mediated regulation AR formation**.

**Category**	**Putative function**	**SEQ_ID**
Auxin mobilization	IAA-AAH	GO_dr001P0019G03_F_ab1
Auxin	GH3	GO_drpoolB-CL6160Contig1
conjugation	GH3	cn5291
	GH3	GO_drpoolB-CL42Contig2
	GH3	cn6745
Auxin transport	Auxin efflux carrier	DC244394_1
	Auxin efflux, PIN-like	GO_drpoolB-CL4639Contig1
	Auxin influx transport	GO_drs31P0009M18_F_ab1
	PINOID	GO_dr004P0019E05_F_ab1
	PINOID-binding	GO_drpoolB-CL3499Contig1
Auxin-binding	Auxin-binding germin	GO_dr004P0021L02_F_ab1
Auxin-induced, protein modification	Ubiquitin-protein ligase	GO_drpoolB-CL5118Contig1
Auxin-induced,	Aux/IAA	GO_drpoolB-CL3347Contig1
transcriptional	Aux/IAA	GO_drpoolB-CL8489Contig1
repressor	Aux/IAA	GO_drpoolB-CL9284Contig1
	Aux/IAA	cn5900
Auxin-regulated,	ARF	GO_drpoolB-CL1607Contig1
transcription	ARF	GO_drpoolB-CL5029Contig1
factor	ARF	IP_PHBS009M22u
Auxin-induced,	SAUR-family protein	cn10015
different functions	SAUR-family protein	cn7580
Ethylene	ACS	GI_TC1166
biosynthesis	ACS	IP_PHBS001D24u
	ACO	cn1774
	ACO	cn3506
Ethylene	ERF	cn2811
responsive,	ERF	GO_drpoolB-CL1692Contig1
transcriptional	ERF	GO_drpoolB-CL2213Contig1
regulation	ERF	GO_dr004P0020P19_F_ab1
Ethylene responsive	LEA-like	cn9245

*Presented is the putative function and SEQ_ID according to Table S1d*.

## Author contributions

Conceived and designed the microarray experiment: Uwe Druege, Philipp Franken, Bettina Hause, Mohammad R. Hajirezaei. Performed the experiments: Uwe Druege, Sandra Lischewski, Siegfried Zerche. Analyzed the data: Philipp Franken, Uwe Druege, Sandra Lischewski, Amir H. Ahkami, Bettina Hause. Developed the model: Uwe Druege. Wrote the paper: Uwe Druege, Philipp Franken. Edited the manuscript: Bettina Hause, Mohammad R. Hajirezaei. Principal investigator: Uwe Druege.

### Conflict of interest statement

The authors declare that the research was conducted in the absence of any commercial or financial relationships that could be construed as a potential conflict of interest.
