# Supplementary material for: Transcriptomic analysis reveals ethylene as stimulator and auxin as regulator of adventitious root formation in petunia cuttings
Source: Front Plant Sci. 2014 Sep 26;5:494. doi: 10.3389/fpls.2014.00494 (PMC4212214; doi:10.3389/fpls.2014.00494)
Supplement: Supplementary file 2 [file Table2.PDF]

Table S2 | Direction of response of AR formation-regulated genes of the auxin and ethylene biosynthesis and signal perception pathways. Based on number of up- versus down-regulated genes presented in Tables 2 and 3 an on the intensity of regulation presented in Table S1. Red and green boxes: preferential up- and down-regulation, respectively, pink boxes: marginal up-regulation, yellow boxes: no regulation or balanced up- versus down-regulation among different genes. Mo: meristemoids, Me: root meristems, Pr: Root primordia, Ro: fully developed roots

Category	Sub-category	2 hpe	6 hpe	24 hpe	72 hpe	96 hpe	144 hpe	196 hpe
Auxin metabolism	Synthesis: YUCCA	[green]	[green]	[green]	[green]	[green]	[green]	[green]
	Mobilization: IAA-AAH	[red]	[green]	[green]	[green]	[green]	[green]	[green]
	Conjugation: GH3-like	[red]	[red]	[red]	[red]	[red]	[red]	[red]
Auxin transport	Influx	[green]	[green]	[yellow]	[yellow]	[red]	[yellow]	[yellow]
	Efflux	[green]	[yellow]	[red]	[green]	[yellow]	[green]	[green]
	Pinoid	[green]	[green]	[green]	[green]	[yellow]	[yellow]	[green]
	Pinoid-binding	[red]	[red]	[red]	[red]	[red]	[red]	[red]
	germin	[green]	[green]	[green]	[green]	[green]	[green]	[green]
Auxin perception	TIR/AFB complex	[green]	[green]	[green]	[green]	[yellow]	[green]	[green]
	Auxin/IAA family	[red]	[green]	[red]	[green]	[green]	[red]	[green]
	ARF	[green]	[green]	[green]	[green]	[green]	[green]	[green]
Ethylene synthesis	ACC synthase	[red]	[red]	[red]	[red]	[red]	[red]	[red]
	ACC oxidase	[red]	[red]	[red]	[red]	[red]	[red]	[red]
Ethylene reception and signaling	Receptors	[yellow]	[pink]	[pink]	[yellow]	[yellow]	[yellow]	[yellow]
	EIN3	[yellow]	[yellow]	[pink]	[yellow]	[yellow]	[yellow]	[yellow]
	ERFs	[red]	[red]	[red]	[red]	[red]	[red]	[red]
	ER co-activator	[yellow]	[yellow]	[red]	[pink]	[red]	[red]	[yellow]
Root development	stages				**Mo**	**Me**	**Pr**	**Ro**
