# Supplementary material for: Transcriptomic analysis reveals ethylene as stimulator and auxin as regulator of adventitious root formation in petunia cuttings
Source: Front Plant Sci. 2014 Sep 26;5:494. doi: 10.3389/fpls.2014.00494 (PMC4212214; doi:10.3389/fpls.2014.00494)
Supplement: Supplementary file 3 [file Presentation1.PDF]

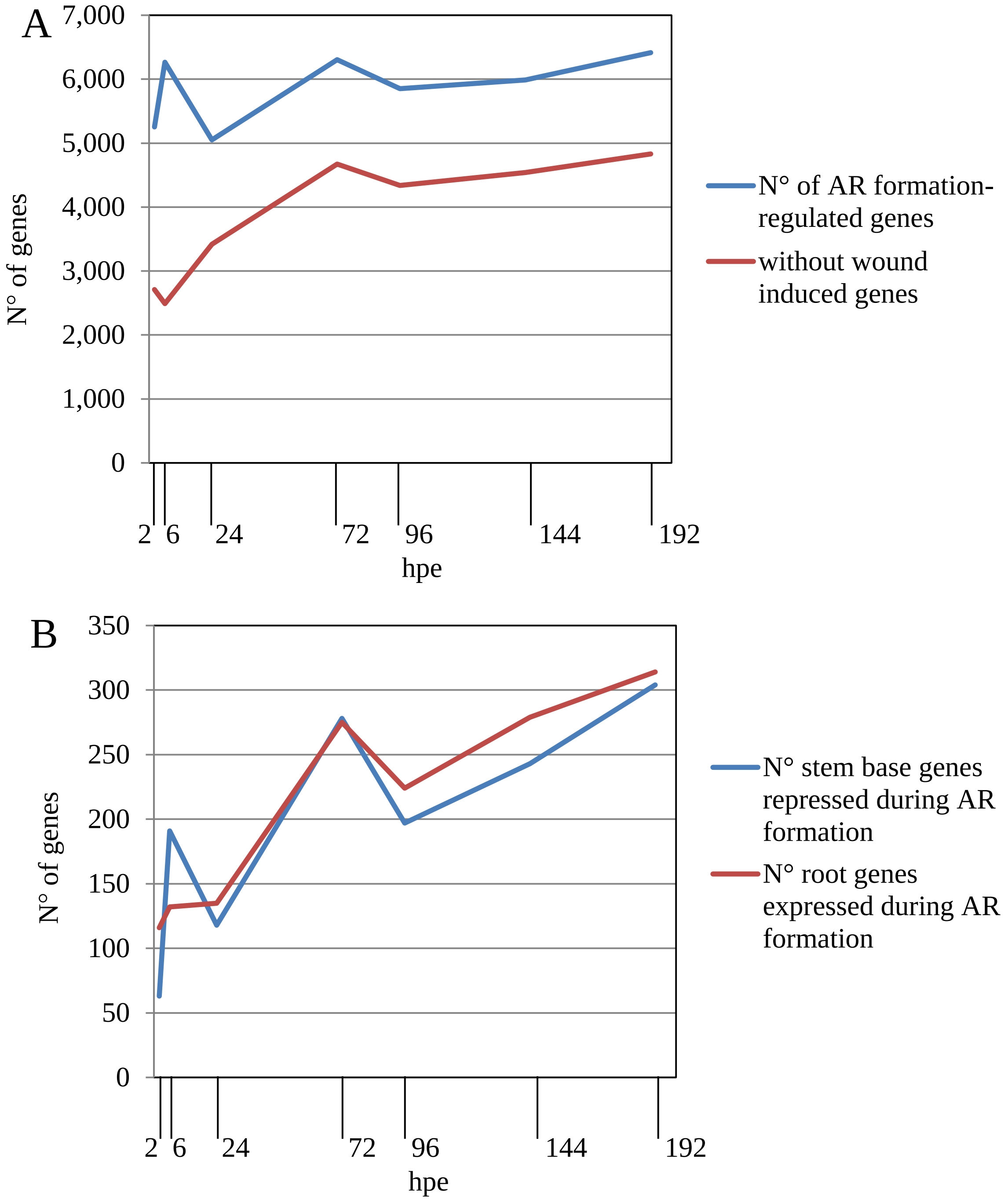

Figure S1 │Gene expression patterns. Number of genes regulated during AR formation at different hours post excision (hpe) compared to stem base prior to excision (A) and number of repressed stem base genes and expressed roots genes (B). Figure is based on the data of Table 1.
